# Dr. Daniel H. Foster

**Published:** 1915-06

**Authors:** 


**Dr. Daniel H. Foster,** Eclectic of Cincinnati 1874, died at his home in Scottsburg, Jan. 31, aged 79.

